# Findings of Multiple Myeloma in Afro-Caribbean Patients in the United States

**DOI:** 10.1200/JGO.17.00133

**Published:** 2018-02-08

**Authors:** Ashtami Banavali, Elvira Neculiseanu, Padma L. Draksharam, Sireesha Datla, Maushmi Savjani, Jennifer Park, Gurinder Sidhu, Evelyn O. Taiwo

**Affiliations:** **Ashtami Banavali**, **Elvira Neculiseanu**, **Padma L. Draksharam**, **Sireesha Datla**, **Maushmi Savjani**, **Jennifer Park, Gurinder Sidhu**, and **Evelyn O. Taiwo**, State University of New York Downstate; and **Evelyn O. Taiwo**, Kings County Hospital, Brooklyn, NY.

## Abstract

**Background:**

Multiple myeloma (MM) is the second most common malignancy in the United States and has a higher incidence in the black and Afro-Caribbean population. There remain limited data on disease presentation and clinical characteristics in this patient group in the United States. The clinical profile of MM in this underrepresented patient group is described here.

**Methods:**

This retrospective study was conducted at Kings County Hospital, an urban New York City hospital in a majority Afro-Caribbean neighborhood. Data from patients diagnosed with MM from 2000 through 2013 were collected from the institution’s tumor registry. Clinical and demographic characteristics of these patients were then analyzed.

**Results:**

Patients with a diagnosis of MM were identified (N = 287). Data were available for 231 patients and of these, 97% self-identified as black. 55% were female, and there was a male-to-female ratio of 1:1.2. The mean age of female patients was 64 years; that of male patients was 63 years. Of the 231 patients, 81% had anemia, 68% had bone lesions, 47% had renal impairment, and 29% had hypercalcemia. Low levels of monoclonal protein were present in 27% of patients and 57% had disease of International Staging System stages I and II. Women had higher BMI than men.

**Conclusion:**

The mean age of presentation of MM in Afro-Caribbean patients is similar to that in the standard population; however, unlike the general US population, there was a higher incidence in women; mean BMI of women also was higher than that of male patients. A sizeable percentage of Afro-Caribbean patients with MM presented with low levels of monoclonal protein in the presence of multiorgan involvement and damage, suggesting the need for early and aggressive diagnostic testing.

## INTRODUCTION

Factors associated with the higher incidence of multiple myeloma (MM) among the black patients compared with white patients in the United States remain unknown. The disease accounts for 10% of all hematologic malignancies and analysis of SEER data on approximately 35,000 patients with MM over 32 years found the disease is two to three times more common in black patients than their white counterparts.^1^ Medical literature on MM incidence, presentation, and survival in AA is limited, although the decreased survival in these patients is well established. A 2003 study revealed younger age of onset and higher MM-specific mortality rates in black patients,^2^ with growing evidence suggesting that MM is a biologically different disease with varied outcomes.^2,3^

Publications and discussions about disease incidence, prevalence, survival, and disparity between black people and white people generally group all patients of African descent in the same category at the expense of genetic differences that may exist within people of African descent. When possible, it is important to study these groups separately to observe the absence or presence of disparity within patient groups.

Our institution, Kings County Hospital, is a municipal hospital located in Brooklyn, a New York City borough with the highest population of Afro-Caribbean patients in the city. According to American Community Survey data for 2010 through 2014, Kings County, New York, was ranked second with respect to Caribbean immigrant population after Dade County in Miami, Florida, with about 21% of Caribbean foreign-born US residents residing in New York City.^4-7^ The demographics of our patient population allow us to study a patient population that, for clinical studies and trial purposes, is traditionally labeled and grouped as African American. Of the patients served by the hospital, 92% are non-Hispanic black and most of our patients are of Afro-Caribbean origin.

Studies have confirmed the higher incidence of MM in black patients. There are 15.1 cases per 100,000 men compared with 7.5 in the general male population and 11.2 per 100,000 women compared with 4.5 in the general female population.^8^ Nossent et al^9^ showed that the incidence of MM in a single French West Indies medical institution was the highest among all hematologic malignancies.^9^ In their study of MM at the University Hospital of West Indies, Buchner-Daley et al^10^ found that most patients with MM had the most advanced stage and end-organ damage. Contrary to reported findings in the US population, Buchner-Daley et al^10^ found that the profile of the Caribbean patients in their study was similar to findings in the white population in the United States. It remains unclear if the clinical characteristics of MM are different in Afro-Caribbean patients who reside in the West Indies compared with the Afro-Caribbean population in the United States.

Review of the tumor registry at our hospital also confirmed the higher-than-average incidence of MM. There remains little information on the disease in the Afro-Caribbean population in the United States, and because of the demographics of our hospital, we are able to describe the clinical profile of the disease in these patients.

## PATIENTS AND METHODS

This is a retrospective study of patients diagnosed with MM at Kings County Hospital from the year 2000 through 2013. Patients were identified and their clinical profiles were collected from the tumor registry and electronic medical records.

A diagnosis of active MM was made on the basis of International Myeloma Working Group (IMWG) criteria, which define active MM as ≥ 10% clonal bone marrow plasma cells, or biopsy-proven bone or extramedullary plasmacytoma in the presence of at least one of the following markers of end-organ damage: hypercalcemia, renal dysfunction, anemia, or bone lesions, referred to, as a group, as CRAB. Regardless of the presence or absence of end-organ damage, the updated IMWG also defines as active MM the presence of one or more of the following: ≥ 60% clonal bone marrow plasma cells, involved-to-uninvolved serum free light chain ratio of ≥ 100, or more than one focal lesion ≥ 5mm on magnetic resonance imaging. Patients with any of these findings were included in the analysis.

Patients with a serum monoclonal protein (M-protein) level < 3g/dL, clonal plasma cells < 10% in the absence of end-organ damage, and plasmacytoma were diagnosed with monoclonal gammopathy of unknown significance (MGUS) and were excluded. Patients with serum M-protein (immunoglobulin [Ig] G or IgA) levels ≥ 3g/dL, urine monoclonal protein level > 500 mg/24 hours, and/or 10% to 60% clonal bone marrow plasma cells in the absence of end-organ damage were diagnosed as having smoldering MM and were excluded, as were patients with solitary plasmacytomas.

Laboratory parameters, including quantity of M-protein and IgG, IgA, or IgM; type of M-protein; serum free light chains and ratio; monoclonal plasma cell percentage in the bone marrow; and International Staging System (ISS) stage were recorded. The presence of CRAB and cytogenetic abnormalities were documented. Epidemiologic parameters (ie, age, sex, and race) were also obtained.

Continuous data were analyzed and are presented as mean ± standard deviation; categorical data are presented as percentages. Comparison between the groups for continuous variables was performed using Student *t* test and χ^2^ or Fisher exact test was used for the categorical variables. *P* < .05 was considered statistically significant. All statistical analyses were performed using MedCalc Software, version 15.8 (Ostend, Belgium).

## RESULTS

A total of 287 patients with a diagnosis of MM were identified. Incomplete charts were excluded and data from 231 patients were analyzed (Table 1). Of the 231, 224 patients (97%) self-identified as black. There were more women (n = 128; 55%) than men (n = 103; 45%); the male-to-female ratio was 1:1.24. Although most patients (n = 169; 73%) had M-protein levels ≥ 3g/dL and/or urine monoclonal protein levels ≥ 500 mg/24 hours, 62 patients (27%) had M-protein levels < 3 g/dL and were labeled as hyposecretory. Of the identified hyposecretory patients, 38 (61%) were women and 24 (39%) were men; the male-to-female ratio was 1:1.56.

**Table 1:**
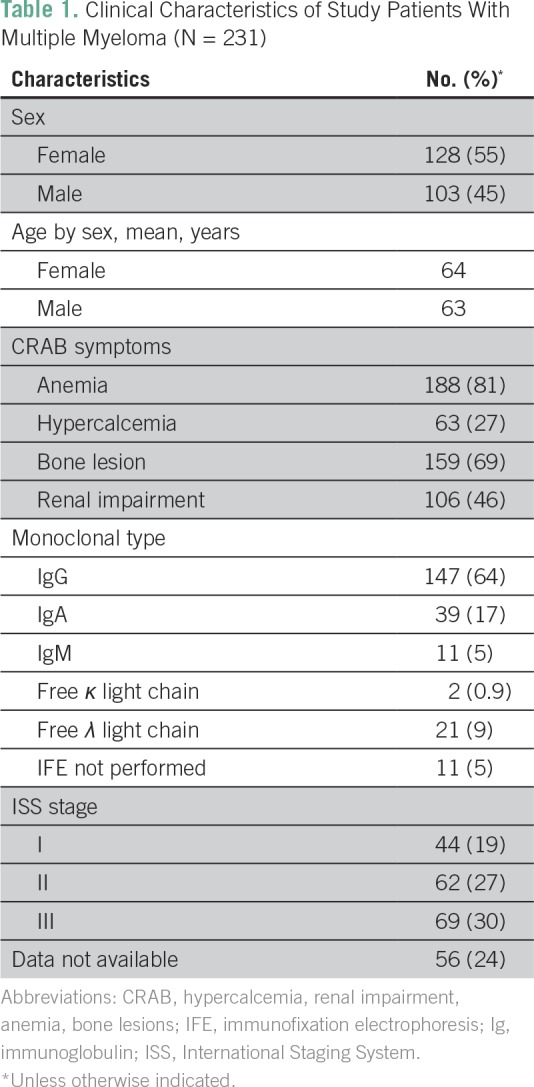
Clinical Characteristics of Study Patients With Multiple Myeloma (N = 231)

Anemia, present in 82% of the hyposecretory group and 79% of all other patients, was the most common presenting clinical feature. IgG-κ was the most common monoclonal protein in all patients. The mean plasma cell percentage in the bone marrow was 40% in the hyposecretory group compared with 51% in the standard group. Of the patients in the hyposecretory group, 29% had plasma cell percentage ≥ 60%. There was significantly less κ light chain MM in the hyposecretory group (20% *v* 1%; *P* = .04).

Presence of end-organ disease symptoms was similar in the standard and hyposecretory patient groups, with 80% presenting with anemia, 26% with hypercalcemia, 70% with bone lesions, and 45% with renal impairment (Table 2). Mean age at diagnosis was 64 years and 63 years in women and men, respectively (Table 3). Women had a higher body mass index (BMI) in all patient groups (Table 4).

**Table 2:**
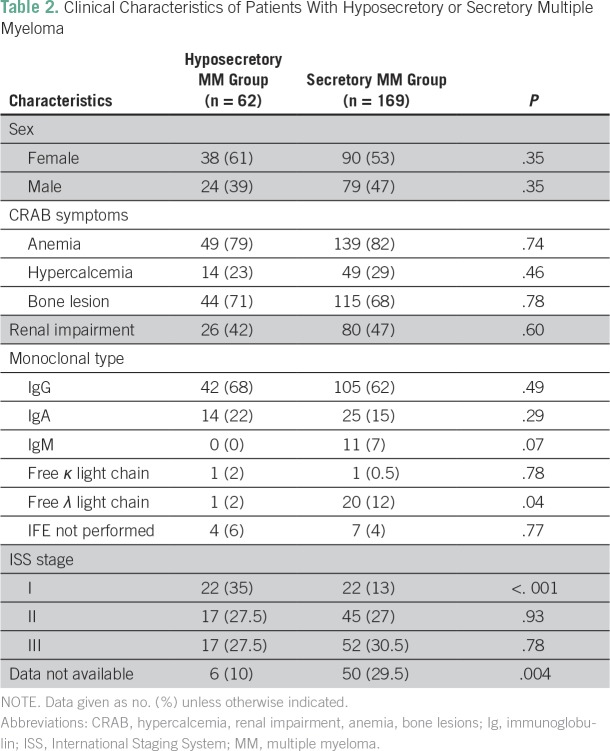
Clinical Characteristics of Patients With Hyposecretory or Secretory Multiple Myeloma

**Table 3:**
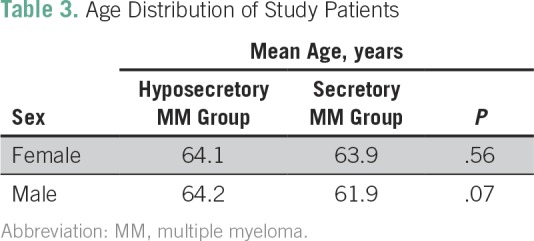
Age Distribution of Study Patients

**Table 4:**
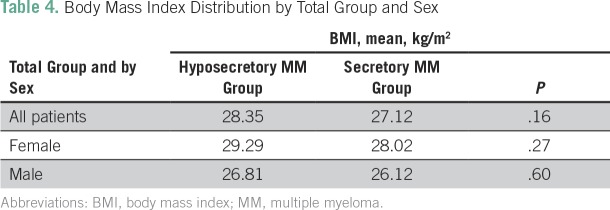
Body Mass Index Distribution by Total Group and Sex

## DISCUSSION

Current knowledge of the clinical characteristics and presentation of MM in the United States has been based on data gathered mostly from white patients. There remain limited data on the demographics and clinical profile of black patients, and more so with black immigrants in the United States who are generally classified as non-Hispanic black. To the best of our knowledge, our study is the largest of its kind to review and analyze the clinical profile and presentation of Afro-Caribbean patients with a diagnosis of MM in the United States.

In our study, a substantial number of Afro-Caribbean patients with low levels of serum monoclonal Ig without the presence of Bence-Jones protein were diagnosed with active MM. Historically, these patients would have been classified as MGUS and a bone marrow biopsy would have been necessary to make a diagnosis. Furthermore, of these patients whom we classified as having hyposecretory MM, 29% had plasma cell percentage ≥ 60%, which, according to the IMWG, qualifies for a diagnosis of active MM requiring treatment. Because current guidelines do not recommend diagnostic bone marrow biopsy procedures be performed in patients with MGUS, it is likely that many Afro-Caribbean patients are diagnosed with active MM later in the course of the disease, when end-organ damage has already occurred.

As shown in Table 2, there were no significant differences in disease stage, presentation of symptoms, and end organ damage in secretory versus hyposecretory MM, which suggests a similar disease course and, therefore, the need to treat both groups similarly.

Contrary to the well-established data also confirmed by the Buchner et al study^10^ in Caribbean patients in the West Indies, in our study, there was a higher incidence of MM in women than men (male-to-female ratio of 1:1.24 compared with 1.54:1 in the general population). In the United States, there is a higher incidence of obesity in the black population, with a 37.1% obesity rate in men compared with 32% in white men and 56.6% in black women compared with 32.8% in white women.^11,12^ The link between obesity and cancer risk was established by a meta-analysis^13^ showing strong evidence of an association of obesity with increased risk of MM overall. That study also showed mortality strongly correlated with BMI.^13^ The higher mean BMI of the female patients in our study compared with that of the men may explain the higher incidence of MM in our patient group. Although our study findings support current evidence that higher BMI, among other causes, is a contributing factor to MM in black patients in the United States^14,15^_,_ it is likely that there are many factors, including genetic differences contributing to the higher incidence of the disease in black patients.

At our hospital, all black patients are classified as non-Hispanic black without distinctions among the patients’ ethnicities. Even though 97% of the patients in our study were black and our hospital primarily caters to patients from the Caribbean and of Afro-Caribbean descent, the inability to subclassify the different groups of black patients in our study limited our ability to compare these patients with the general US patient groups. Data on important factors such as cytogenetic and molecular abnormalities, which are predictive of outcome, were unavailable for many patients and, therefore, were not included in our analysis.

Our study shows that the mean age at diagnosis and type of monoclonal protein (ie, IgG) were similar to findings in the general population. On the other hand, unlike the general population, there was a substantial number of patients presenting with low levels of serum M-protein who were diagnosed with active MM. This finding highlights the importance of aggressive and early diagnostic testing, including bone marrow biopsy, in patients with MGUS. Early diagnosis may prevent disease progression and end-organ damage in the Afro-Caribbean population.
